# Adolescent Self-Efficacy for Diet and Exercise Following a School-Based Multicomponent Lifestyle Intervention

**DOI:** 10.3390/nu14010097

**Published:** 2021-12-27

**Authors:** Vasiliki Efthymiou, Evangelia Charmandari, Dimitrios Vlachakis, Artemis Tsitsika, Artur Pałasz, George Chrousos, Flora Bacopoulou

**Affiliations:** 1 University Research Institute of Maternal and Child Health and Precision Medicine, and UNESCO Chair in Adolescent Health Care, School of Medicine, National and Kapodistrian University of Athens, Aghia Sophia Children’s Hospital, 11527 Athens, Greece; vikimiou2003@yahoo.gr (V.E.); chrousos@gmail.com (G.C.); 2 Division of Endocrinology, Metabolism and Diabetes, First Department of Pediatrics, School of Medicine, National and Kapodistrian University of Athens, Aghia Sophia Children’s Hospital, 11527 Athens, Greece; evangelia.charmandari@googlemail.com; 3 Division of Endocrinology and Metabolism, Center for Clinical, Experimental Surgery and Translational Research, Biomedical Research Foundation of the Academy of Athens, 11527 Athens, Greece; 4 Laboratory of Genetics, Department of Biotechnology, School of Applied Biology and Biotechnology, Agricultural University of Athens, 11855 Athens, Greece; dimvl@aua.gr; 5 MSc “Strategies of Developmental and Adolescent Health”, 2nd Department of Pediatrics, “P. & A. Kyriakou” Children’s Hospital, School of Medicine, National and Kapodistrian University of Athens, 11527 Athens, Greece; info@youth-health.gr; 6 Department of Histology, School of Medical Sciences in Katowice, Medical University of Silesia, 40-752 Katowice, Poland; apalasz@sum.edu.pl; 7 Center for Adolescent Medicine and UNESCO Chair in Adolescent Health Care, First Department of Pediatrics, School of Medicine, National and Kapodistrian University of Athens, Aghia Sophia Children’s Hospital, 11527 Athens, Greece

**Keywords:** self-efficacy, diet, exercise, adolescents, Greece, lifestyle, intervention, KIDMED

## Abstract

Self-efficacy is perhaps the most important parameter associated with behavioral changes. The main aim of this study was to provide insight into the diet and exercise self-efficacy of Greek adolescents and how they could be modified via a multilevel multicomponent school-based lifestyle intervention. Secondary aims were to study the associations of students’ dietary and exercise self-efficacy indices with their anthropometric and sociodemographic parameters. A representative sample of the adolescent population in Attica, consisting of 1610 adolescents aged 12–17 years, recruited from 23 public high schools in three municipalities of the Attica area in Greece, received a three-component lifestyle educational intervention for health promotion and underwent screening for characteristics of metabolic syndrome with the use of portable telemedicine. All assessments and anthropometric measurements were performed at baseline and after the 6-month intervention. Anthropometric measurements included body mass index, waist circumference (WC), waist-to-height ratio (WHtR) and waist-to-hip ratio (WHR). Assessment tools included the Self-efficacy for Diet and the Self-efficacy for Exercise questionnaires, as well as the Mediterranean Diet Quality Index in Children and Adolescents (KIDMED). Analysis included 1020 adolescent students (421 males and 599 females), who completed the self-efficacy questionnaires pre- and post-intervention. Overall, the dietary (*p* < 0.001) and exercise (*p* < 0.001) self-efficacy increased significantly post-intervention. Post-intervention, all adolescents decreased their abdominal obesity indices (WC, WHtR, WHR), and this improvement was even more pronounced and significant (*p* = 0.019, *p* = 0.019, *p* = 0.023 respectively) in the adolescents with overweight/obesity. Post-intervention, the proportion of adolescents with normal weight increased from 73.9% to 78.6%, whereas the proportion of adolescents with overweight and obesity decreased from 20.4% to 15.9% and from 5.7% to 5.5%, respectively. Abdominal obesity also decreased from 10.4% to 9.0%. Female adolescents achieved significantly (*p* = 0.010) higher changes in diet self-efficacy than males. Other sociodemographic characteristics such as family structure, parental age, parental educational level and family income showed non-significant differences. Adolescents with higher KIDMED scores manifested significantly higher dietary and exercise self-efficacy than those with lower KIDMED scores. Both adolescents with normal weight and overweight/obesity manifested a reciprocal relation between diet and exercise self-efficacy. Multicomponent lifestyle interventions in the school environment may provide a first step in students’ behavior changes and provide grounds for future prevention programs in youth.

## 1. Introduction

The concept of self-efficacy was instituted in 1977 by A. Bandura in the context of social cognitive theory and is perhaps the most important parameter associated with behavioral changes [1]. The perception of self-efficacy refers to the capacity to successfully perform a task [2]. The establishment of self-efficacy depends on a dynamic interaction among psychological, physiological and environmental influences [3]. In adolescence, self-efficacy has been related to positive outcomes, i.e., enhanced academic performance, better emotional health and improved quality of life [4,5,6].

The important role of interventions targeting adolescent self-efficacy has been shown in several studies; a randomized controlled trial in Iranian adolescent girls demonstrated significantly improved nutritional behaviors and psychological variables such as self-efficacy in the intervention vs. the control group [7]. In another study, a cooking intervention improved cooking self-efficacy, positive cooking attitude, as well as the quality of the diet and mental well-being of adolescents [8]. Diet self-efficacy is considered an important conceptual element in the development of prevention and treatment strategies for youth eating disorders [9] and for healthier eating habits in general. Diet self-efficacy has been linked to reduced fat and sodium intake and reduced food consumption at mealtimes [10,11,12].

Exercise self-efficacy is another important element to consider in physical activity (PA), with several studies reporting a strong association [13,14,15]. A study of a cohort of inactive participants who participated in an exercise program revealed exercise self-efficacy to be the only significant predictor of exercise maintenance after 9 months follow-up [16]. Another study of preadolescents aged 8–12 years who had an elevated body mass index (BMI), showed that interventions that increase self-efficacy for PA may improve activity levels [17]. Furthermore, there is a stronger association between physical self-perception and self-esteem at higher vs. lower levels of self-efficacy for PA [18]. Another study in previously healthy adult men demonstrated that low exercise self-efficacy was associated with a significantly higher incidence of cardiovascular incidents, after controlling for cardiovascular risk factors [19].

Technology-assisted educational interventions regarding nutrition that have been delivered to adolescents with overweight or obesity had a positive impact on cooking practices in their family home and future dwelling places [20]. An Iranian study has shown that a cognitive-behavioral treatment contributed to the adoption of healthy nutritional habits, a decrease in body fat markers (waist circumference, BMI, waist-hip ratio and fat mass) and an improvement in psychosocial health, PA and health-related quality of life in adolescents [21]. A community pilot study on the group management of obesity has shown that changes in diet and exercise knowledge and self-efficacy are associated with healthy eating [22].

Several studies have found that self-efficacy is the main determinant of exercise maintenance even though it predicts short-term exercise initiation (up to half a year) [23,24,25]. This is consistent with theoretical models about behavioral changes, which imply that the influence of self-efficacy is considerable from the initiation to the maintenance of a behavior [26].

The main aim of this study was to provide insight into the diet and exercise self-efficacy of Greek adolescents and how they could be modified via a multilevel, multicomponent school-based lifestyle intervention. Secondary aims were to study the associations of students’ dietary and exercise self-efficacy indices with their anthropometric and sociodemographic parameters. It was hypothesized that the intervention would enhance adolescents’ self-efficacy for diet and exercise.

## 2. Materials and Methods

The study was part of a European-Union-funded “program for health promotion, prevention and screening for characteristics of metabolic syndrome in adolescents attending high schools in three municipalities (Palaio Faliro, Aghios Dimitrios, Alimos) in the Attica region in Greece, with the use of portable telemedicine”, as described in previous studies [27,28,29]. Adolescents were screened in the school setting for general and abdominal obesity and other metabolic syndrome characteristics, as well as for their dietary and exercise self-efficacy, at baseline and following a 6-month multi-component, multi-level intervention endorsing healthy nutrition, body image and physical activity.

### 2.1. Participants

A representative sample of the adolescent population in Attica, consisting of 1610 adolescents (680 males and 930 females) 12–17 years old was recruited from 23 public high schools of three municipalities (Alimos, Aghios Dimitrios, Palaio Faliro) in Attica, Greece. Schools were selected randomly to participate in a European-Union-funded program (code MIS 371406) for adolescent health promotion and screening for characteristics of metabolic syndrome via telemedicine, in the context of the National Strategic Reference Framework 2007–2013. The program received ethics approval (reference number 86758/Γ2) from the Uniform Administrative Sector of Primary and Secondary Education of the Hellenic Ministry of Education and Religious Affairs.

Adolescent students and their parents/guardians signed written consent forms to participate in the program, which lasted one year (August 2013 until August 2014), from baseline up to the final measurements.

### 2.2. Multicomponent-Multilevel Intervention

After baseline measurements, adolescents participated in a 6-month lifestyle educational intervention. The intervention involved a multilevel-multicomponent approach that included three components—(i) nutritional habits and adherence to the Mediterranean diet (MD), (ii) systematic PA, (iii) healthy eating behaviors and healthy body image. This multilevel design, addressed not only to adolescents but also to other levels of influence on their health, has been used with promising results for primary prevention in childhood and adolescence [30,31,32]. The program also aimed to train parents, educators and healthcare professionals to assist adolescents to embrace a healthy lifestyle. As such, a website, guidebooks and educational lectures, customized for adolescents, parents, teachers, school and community health professionals, were provided by a multidisciplinary team consisting of a pediatrician specialized in adolescent medicine, a psychiatrist, an exercise physiologist and a registered dietitian. During the 6-month intervention, adolescents took part in 36 interactive sessions (of 2 h each), their parents attended 9 sessions (of 4 h each) and teachers/health professionals attended two-day workshops (of 8 h each), with each component covering a third of the lifestyle education time. All lectures were administered at the schools’ auditoriums during the last academic hours on school days for students and in the afternoons/evenings for parents, schoolteachers and health staff.

### 2.3. Materials

All assessments and measurements were performed at baseline, as well as after 6 months, following the completion of the intervention.

#### 2.3.1. Anthropometric Measurements

A team of two registered nurses and one pediatrician visited schools on pre-determined dates, before the first academic hour, to measure participating students’ weight, height and waist and hip circumferences, after an overnight fast. Adolescents’ weight was measured in minimal clothing with electronic portable scales (Fora w100b, ForaCare Suisse AG, St. Gallen, Switzerland), height (Ht) was measured barefoot with portable stadiometers and waist circumference (WC) and hip circumference were measured with inextensible anthropometric tape. For each participant, measurements of height and waist and hip circumferences were inserted manually into the software database, whereas weight was recorded automatically in the database via the scales’ connection to the telemedicine software vida24 (Vidavo Health Telematics, version 1.0, Thessaloniki, Greece).

#### 2.3.2. Sociodemographic Data

Parents were asked to fill in sociodemographic data forms at home. Sociodemographic questions pertained to personal characteristics (age, educational level, family income, family structure) and their educational level (low–medium if up to 12 school-years or high for university/higher education). Sociodemographic data were inserted manually into the program’s database by the team (pediatrician and two nurses).

#### 2.3.3. Self-Efficacy for Diet

This questionnaire was designed to assess the self-efficacy of adolescents to make specific changes in their diet. Self-efficacy is the confidence that persons feel in making changes in their daily lives or in achieving a specific goal. Self-efficacy is crucial in understanding and modifying health behaviors and, more specifically, eating behaviors. Proper nutrition and weight control to prevent diseases are linked to one’s beliefs. People who believe that are capable of achieving a specific goal can make their own choices, pursue a ‘healthy’ lifestyle and resist temptations. The questionnaire was developed specifically for the European program by one of the program participants, namely, Harokopio University, and was completed online. The questionnaire questions are based on self-efficacy questionnaires for adolescents and adults published in the international literature [3,33,34].

To complete the questionnaire, adolescents are asked to answer 20 different questions about how capable they feel to make specific changes to their diet. The questions cover five different domains: (a) fruits and vegetables (questions 1–5); (b) dining habits (questions 6–10); (c) whole foods/processed foods (questions 11–13); (d) hydration (questions 14–17); and (e) low-nutrient foods (questions 5, 18–20). Next to each question there is a scale from 0 to 7; 0 means “I do not feel capable of adopting this particular behavior”, whereas 7 means “I feel very capable of adopting this particular behavior”. The teenager places a vertical line at any point on the scale that he or she considers to be the answer. A line near 0 corresponds to a reduced capacity, whereas a line near 7 corresponds to an absolute capacity. The score of each question is equal to the numerical distance of the line from zero to the vertical line drawn by the respondent, which is measured using a ruler. The total length of the line is 7 cm. The overall questionnaire score is calculated by summing the scores of the individual questions, ranging from 0 to 140. Zero indicates that the adolescents do not feel capable at all of making specific changes to their diet, and 140 indicates that they feel absolutely capable of making specific changes in their diet. Furthermore, scores for each subscale of the domains mentioned above are extracted. It should be noted that questions assess the ability to change, not the desire to change.

#### 2.3.4. Self-Efficacy for Exercise

Self-efficacy is considered an important predictor of exercise [35], whereas people who exercise on a regular basis report higher self-efficacy in overcoming barriers related to their physical condition than those who exercise sporadically or not at all [36].

The questionnaire was developed specifically for the European program by one of the program participants, namely, Harokopio University, and was completed online. The questionnaire questions are based on different questionnaires on self-efficacy for exercise [33,34,37,38,39] and reflect the recommendations for PA in adolescents, as well as the factors that have been identified to affect adolescents’ PA. Such factors include support from friends or family [40,41], the weather, the ability to participate in organized out-of-school activity, access to places that support PA [42], as well as sedentary tasks on computers, video games and reading [43].

The questionnaire consists of 15 questions, each answered on a scale of 0 to 7, with the participant marking a vertical line at that point of the scale that he or she considers to correspond to their answer. The questionnaire scores are derived from the sum of the scores of the individual questions. The weight of each question is equal to the numerical distance of the line drawn by the respondent from zero, which is measured using a ruler. The tool’s rating ranges from 0 to 105, with higher scores suggesting higher self-efficacy for performing the exercise.

#### 2.3.5. Assessment of Dietary Habits

Students’ eating habits were assessed according to the adherence to a Mediterranean diet (MD), with the use of the Mediterranean Diet Quality Index in Children and Adolescents (KIDMED) [44]. The KIDMED tool is a self-reported questionnaire of 16 items that includes questions regarding healthy (such as vegetables, cereals, fruit, nuts, etc.) or unhealthy (such as sweets, fast foods, etc.) nutritional habits that are assigned a score of +1 or −1 respectively. The total score range is −4 to 12, with scores ≥8 indicating optimal adherence to MD, scores between 4 and 7 suggesting the need for improvement, and scores ≤3 indicating low adherence to MD. The KIDMED Index was validated in the HELENA study in European adolescents of nine European countries, including Greece [45].

### 2.4. Statistical Analysis

Analyses were conducted using SPSS software 22 version for Windows (IBM Statistical Package for Social Sciences for Windows, Version 22.0. Armonk, NY: IBM Corp).

BMI was calculated as the ratio of body weight (kg) to the square of height (m^2^), waist-to-height ratio (WHtR) as the ratio of WC to Ht and waist-to-hip ratio (WHR) was calculated as the ratio of WC to hip circumference. Differences between two timeframes (Δ Diet or exercise self-efficacy) were computed as post-intervention minus pre-intervention (baseline).

Qualitative variables are shown as absolute and relative frequencies (percentages) and continuous variables are shown as mean ± standard deviation (SD). Student’s *t*-test or a one-way analysis of variance (ANOVA), using the Bonferroni correction for multiple comparisons, were used to evaluate associations between continuous variables and several characteristics. Paired *t*-tests were used to evaluate the intervention outcomes. Associations between two continuous variables were conducted using the Pearson correlation. All reported probability values (*p*-values) were established using two-sided tests. The significance level was set at 5%.

## 3. Results

**Figure 1:**
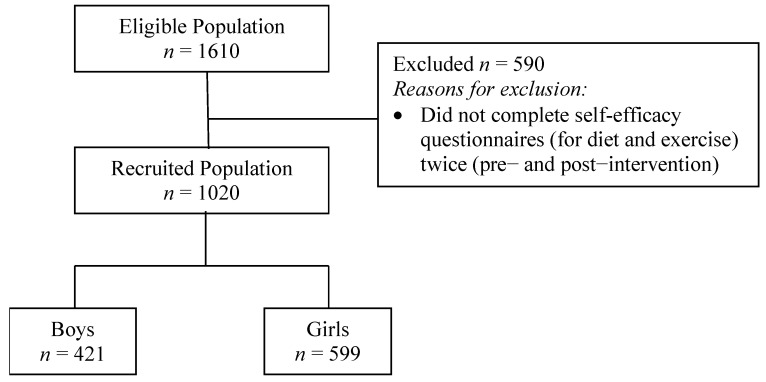
Flowchart of study participants.

Data from 1020 adolescent students who had completed the diet and exercise self-efficacy questionnaires both at baseline (pre-intervention) and post-intervention were included in the analysis, as shown in Figure 1.

Adolescents’ demographic and anthropometric characteristics, pre- and post-intervention, are shown in Table 1. The adolescent sample (males vs. females) was age-matched (*p* = 0.055) with a male-to-female ratio of 0.70 (41.3% males and 58.7% females) and a mean age (±SD) of 14.08 (±1.63) years. According to international cut-offs [46], at baseline, 73.9% of adolescents had a normal weight, 20.4% were overweight, and 5.7% had obesity. The mean (±SD) score for diet self-efficacy increased significantly from 94.21 (±24.04) at baseline to 100.49 (±23.00) post-intervention (*p* < 0.001) and the mean (±SD) score for exercise self-efficacy increased significantly from 82.18 (±25.31) at baseline to 85.25 (±19.80) post-intervention (*p* < 0.001). However, these favorable changes in exercise self-efficacy were non-significant in the participants with overweight/obesity. Overall, statistically significant decreases post-intervention were observed in WC (*p* = 0.007) from 70.97 (±9.14) cm at baseline to 70.52 (±9.04) cm, WHtR (*p* = 0.006) from 0.435 (±0.053) at baseline to 0.432 (±0.052), and WHR (*p* = 0.015) from 0.758 (±0.069) at baseline to 0.753 (±0.065). The statistically significant decreases in these abdominal obesity indices were mainly attributed to the adolescents with overweight/obesity. Post-intervention, mean (±SD) differences in WC, BMI and BMI z-scores were equal to −0.45 (±5.15) cm, 0.43 (±1.01) kg/m^2^ and 0.12 (±0.28), respectively. Considering the anticipated increases in body weight and BMI associated with expected weight gain during adolescence, post-intervention the proportion of adolescents with normal weight increased to 78.6%, whereas the proportion of adolescents with overweight and obesity decreased to 15.9% and 5.5%, respectively. Abdominal obesity, assessed by age- and ethnic-specific percentiles [27], decreased, but not significantly (*p* = 0.117), from 10.4% to 9.0%.

Adolescents’ differences (Δ) in diet and exercise self-efficacy scores were evaluated considering their sociodemographic characteristics (Table 2). Female adolescents achieved a significantly higher change (*p* = 0.010) in diet self-efficacy than male adolescents (mean ± SD 7.82 ± 22.30 vs. 4.10 ± 22.99, respectively). Regarding exercise self-efficacy, females also achieved a higher (*p* = 0.102) change than males. Non-significant differences were observed in other sociodemographic characteristics such as family structure, parental age, parental educational level and family income.

Diet and exercise self-efficacy were evaluated according to the KIDMED scores pre- and post-intervention (Table 3). Adolescents with higher scores exhibited significantly higher diet self-efficacy than those with lower KIDMED scores. The same pattern was observed in exercise self-efficacy, pre- and post-intervention.

**Figure 2:**
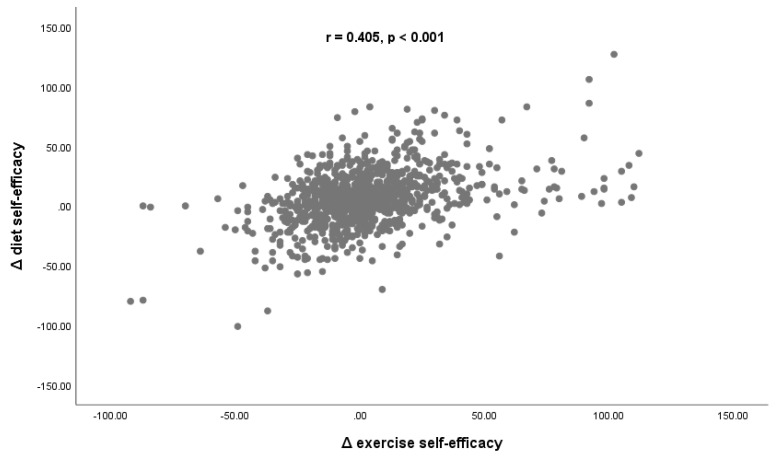
Scatterplot of (Δ) differences (post- vs. pre-intervention) between diet and exercise self-efficacy and their correlation.

The correlation (r = 0.405, *p* < 0.001) between the two target assessments, i.e., diet and exercise self-efficacy, is displayed in Figure 2.

**Figure 3:**
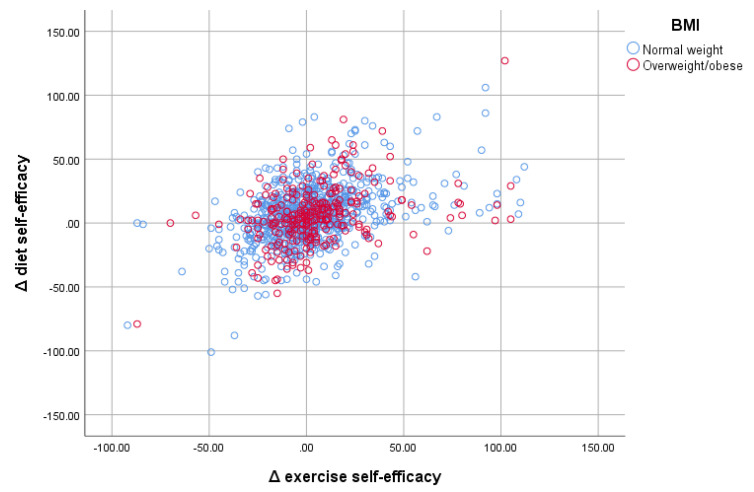
Scatterplot of (Δ) differences (post- vs. pre-intervention) between diet and exercise self-efficacy, presented separately for adolescents with normal weight and overweight/obesity.

Figure 3 presents the scatterplot presenting diet and exercise self-efficacy separately for adolescents with normal weight and overweight/obesity. There was homogeneity in the group with overweight/obesity, with most adolescents demonstrating a difference of approximately 20 points in diet and exercise self-efficacy. On the other hand, the normal-weight group showed greater scattering in their differences post- vs. pre- intervention. Both groups showed significant correlations between diet and exercise self-efficacy.

## 4. Discussion

Proper nutrition and weight control to prevent diseases are linked to personal beliefs. People who believe they are capable of achieving a particular outcome can make their own choices, pursue a healthier lifestyle and resist temptations. This is in line with theoretical models describing mechanisms that facilitate the progress from intervention to behavioral effects [47], but when a behavior is established, inspiration becomes the major determinant of behavior conservation [26]. In our study, there was an improvement in diet and exercise self-efficacy after the multicomponent multilevel lifestyle intervention in these Greek public schools. One could speculate that adolescents’ confidence in making changes in their daily lives was activated and their self-efficacy to modify nutritional and PA behaviors was enhanced. Following intervention, the proportion of normal-weight adolescents increased from 73.9% to 78.6%, whereas the proportions of overweight and obesity decreased from 20.4% and 5.7% to 15.9% and 5.5% respectively. Adolescents’ behavioral changes were accompanied by improvements in their abdominal obesity indices—WC, WHtR, and WHR—post-intervention. These changes were translated into a decrease in adolescents’ abdominal obesity from 10.4% to 9.0%, which, although statistically non-significant, can still be considered as a meaningful outcome in view of the high prevalence of abdominal obesity in Greek adolescents [29,48]. Although beneficial changes in abdominal obesity indices from baseline values were observed for all participants following the intervention, the impact was even more apparent and significant in the adolescents with overweight/obesity.

The enhancement of diet and exercise self-efficacy was investigated according to the sociodemographic characteristics of the sample; only sex was related to diet self-efficacy, with females achieving significantly higher changes in diet self-efficacy than males. In another study, high diet self-efficacy of older female adolescents was linked to the lowest fat and sodium levels [10]. Similar findings have been reported for females ordering fewer dishes with high-calories, whereas males were found to be non-responsive in terms of changing their choices regarding calories, following a technology-based intervention [49]. There are sex differences in the perception of and satisfaction with body weight, with females seeing themselves as being overweight, whereas males are more likely to view themselves as underweight and want to be heavier and more muscular [50].

Several studies have emphasized the critical role of family in self-efficacy to establish behavioral changes and future lifestyle decisions [51,52]. In the present study, lectures to parents were not mandatory to attend and there were no records about how many parents or which parent (father or mother) eventually attended. Nevertheless, school staff urged parents to attend the lectures, at least one from each family. There was a tendency for adolescents from low-income families with less-educated parents to score higher in diet and exercise self-efficacy. Adolescents from poor families may benefit from such interventions more than students from higher sociodemographic backgrounds, who have probably been previously exposed to such stimuli. This finding did not support the hypothesis that adolescents from unfavorable social backgrounds have poor attitudes and knowledge regarding nutrition, and low self-efficacy due to the absence of confidence and a shortage of healthy food [53,54].

In our study, adolescents with higher (vs. lower) adherence to the MD demonstrated significantly higher self-efficacy in regard to their diet. Similarly, another study found that teenagers with proper eating behavior, receiving two or more major meals daily, had significantly higher knowledge and self-efficacy scores than those who had one or fewer major meals daily [55]. In line with our findings, self-efficacy has been found to be a strong predictor of adolescent healthy behavior [56,57]. We also found adolescents’ proper eating habits (evaluated by the KIDMED) to be associated with higher exercise self-efficacy, as nutritional and PA self-efficacy appeared to be interrelated.

Some adolescents experienced unfavorable changes in self-efficacy. A study of 1111 female 15-year-old students in Poland, evaluating the effects of a one-year program on changes in healthy eating and physical activity, revealed a decrease in the general self-efficacy score. In the same study, the group with overweight/obesity demonstrated non-significant changes, whereas a statistically significant decrease was observed in non-overweight girls [58]. Several factors, i.e., psychological, social (culture, family, peer pressure) and physical (body weight) factors, may interact with the effects of interventions on self-efficacy. Furthermore, in the present study, changes in exercise self-efficacy were not significant in the adolescents with overweight/obesity, suggesting that self-efficacy in this population may be not the only mediator to physical activity changes [59]. Furthermore, a smaller increase in activity, compared with their normal-weight peers, might have been sufficient to produce significant effects on their anthropometrics over 6 months.

Limitations of the present study include the absence of long-term follow-up to evaluate the sustainability in the enhancement of adolescents’ nutritional behaviors and physical activity. The non-mandatory parental participation in the intervention did not provide any information as to parental involvement, and peer interaction was not assessed in the context of adolescents’ changes in behavior. Furthermore, the effects on teacher awareness were not studied. Nonetheless, the program was successful in the short term, achieving improved diet and exercise behaviors in a representative sample of the adolescent population in Attica. On the other hand, causative associations cannot be confirmed, but only assumed, as this was a single-arm interventional study without a control group [60]. Furthermore, the lack of validation of the online self-efficacy questionnaires that were developed specifically for the European program by one of the program participants, namely, Harokopio University, constitutes an important limitation of the study. Furthermore, psychological disorders (i.e., depression, low resilience) that may affect and act as barriers to self-efficacy [61,62], were not investigated.

Despite the efforts made, the prevention of obesity in children and adolescents has been elusive so far, with limited effectiveness of intervention programs [63,64]. Our study underlines the significance of school-based lifestyle interventions in the enhancement of adolescents’ dietary and PA behaviors and the improvement of adolescents’ anthropometric indices related to obesity. It also provides proof on the linkage between dietary and PA habits. Findings from the present study are in line with the results of a recent systematic review providing comprehensive insight into the need for a health-promoting multi-level (home, environment, school, peers) approach to influence lifestyle habits in children and adolescents [65].

The foundation for health is laid at a young age. Adolescents spend most of their time at home and at school. Lifestyle programs based on a strict sequence of activities and teaching, as well as on the sense of interaction and coherence in schools, may promote nutrition and activity messages without the need for many added resources, and may improve students’ self-efficacy for healthy behaviors. Schools should become an integral part of the battle against the obesity epidemic and targeting self-efficacy may lower the risk for obesogenic behaviors. Multicomponent lifestyle interventions in the school environment may establish the first step in students’ behavioral changes and provide grounds for future well-designed, large-scale longitudinal prevention studies in youth.

## Figures and Tables

**Table 1:** Adolescents’ demographic and anthropometric characteristics pre- and post-intervention (N = 1020) and according to weight status.

	Total Sample			Normal Weight			Overweight/Obesity		
	Intervention		*p* ^‡^	Intervention		*p* ^‡^	Intervention		*p* ^‡^
	Pre	Post		Pre	Post		Pre	Post	
Males (%) ^†^	421 (41.3)			285 (38.5)			124 (47.5)		
Age (years)	14.08 ± 1.63			14.17 ± 1.63			13.84 ± 1.62		
WC (cm)	70.97 ± 9.14	70.52 ± 9.04	**0.007**	67.39 ± 5.54	67.16 ± 5.72	0.166	81.08 ± 9.59	80.02 ± 9.81	**0.019**
WHtR	0.435 ± 0.053	0.432 ± 0.052	**0.006**	0.413 ± 0.030	0.412 ± 0.032	0.140	0.496 ± 0.056	0.489 ± 0.057	**0.019**
WHR	0.758 ± 0.069	0.753 ± 0.065	**0.015**	0.745 ± 0.061	0.741 ± 0.056	0.141	0.796 ± 0.078	0.787 ± 0.077	**0.023**
BMI (kg/m^2^)	21.38 ± 3.54	21.81 ± 3.62	**<0.001**	19.77 ± 1.97	20.25 ± 2.07	**<0.001**	25.95 ± 2.98	26.23 ± 3.42	**<0.001**
BMI z-score	0.47 ± 0.86	0.59 ± 0.82	**<0.001**	0.09 ± 0.64	0.25 ± 0.62	**0.005**	1.54 ± 0.36	1.55 ± 0.43	**0.001**
Diet self-efficacy score	94.21 ± 24.04	100.49 ± 23.00	**<0.001**	93.90 ± 23.79	100.22 ± 23.07	**<0.001**	95.92 ± 24.35	102.50 ± 21.92	**0.001**
Exercise self-efficacy score	82.18 ± 25.31	85.25 ± 19.80	**<0.001**	82.94 ± 24.74	85.44 ± 19.80	**<0.001**	79.72 ± 26.75	84.93 ± 19.82	0.170

Values are displayed as means ± SD or ^†^ frequencies (percentages). ^‡^ *p*-value of paired *t*-test. Bold indicates statistically significant differences. WC, waist circumference; WHtR, waist-to-height ratio; WHR, waist-to-hip ratio; BMI, body mass index.

**Table 2:** Sociodemographic characteristics of adolescents according to differences in diet and exercise self-efficacy scores.

	Δ Diet Self-Efficacy ^§^		Δ Exercise Self-Efficacy ^§^	
	Mean ± SD	p *	Mean ± SD	p *
Sex				
Male	4.10 ± 22.99	**0.010**	1.57 ± 23.48	0.102
Female	7.82 ± 22.30		4.13 ± 25.46	
Family structure				
Two parents	6.63 ± 22.28	0.470	2.76 ± 24.28	0.157
One parent	8.22 ± 25.08		6.68 ± 28.97	
Maternal age				
30–49	6.82 ± 22.90	0.593	2.93 ± 25.20	0.747
50+	7.96 ± 20.91		3.69 ± 23.52	
Paternal age				
30–49	7.17 ± 22.55	0.544	3.78 ± 26.02	0.403
50+	6.24 ± 22.45		2.35 ± 23.41	
Maternal educational level				
Low-Medium	7.53 ± 23.37	0.338	3.95 ± 25.90	0.395
High	6.12 ± 21.88		2.57 ± 23.97	
Paternal educational level				
Low–medium	7.36 ± 22.40	0.455	4.14 ± 24.98	0.257
High	6.25 ± 22.93		2.29 ± 24.93	
Family income				
<10,000 EUR	7.45 ± 22.65	0.093	3.55 ± 24.76	0.487
10,001+ EUR	4.35 ± 22.53		2.14 ± 25.78	

Values are displayed as means ± SD. ^§^ Δ Diet and exercise self-efficacy refer to the difference between post-intervention and baseline (pre-intervention). * *p*-value of Student’s *t*-test. Bold indicates statistically significant differences.

**Table 3:** Diet and exercise self-efficacy according to KIDMED scores, at baseline (pre-) and post-intervention.

KIDMED Score	Diet Self-Efficacy				Exercise Self-Efficacy			
	Baseline	*p*	Post Intervention	*p*	Baseline	*p*	Post Intervention	*p*
≥8	104.97 ± 21.44	**<0.001 ^‡,a,b,c^**	112.27 ± 17.64	**<0.001 ^‡,a,b,c^**	87.58 ± 25.76	**<0.001 ^‡,a,b^**	92.57 ± 16.11	**<0.001 ^‡,a,b,c^**
4–7	94.17 ± 22.47		99.79 ± 21.57		81.46 ± 25.15		84.17 ± 19.97	
<3	81.32 ± 25.61		85.51 ± 25.83		77.57 ± 23.76		78.39 ± 21.14	

Values are displayed as means ± SD. ^‡^ *p*-value of analysis of variance (ANOVA). Post hoc comparisons using the Bonferroni test between ^a^ “≥8” and “4–7”, ^b^ “≥8” and “≤3”, ^c^ “4–7” and “≤3” categories. Bold indicates statistically significant differences.

## Data Availability

Data available on request.
